# Relationship between Total Antioxidant Capacity, Cannabinoids and Terpenoids in Hops and Cannabis

**DOI:** 10.3390/plants12061225

**Published:** 2023-03-08

**Authors:** Philip Wiredu Addo, Zohreh Poudineh, Michelle Shearer, Nichole Taylor, Sarah MacPherson, Vijaya Raghavan, Valérie Orsat, Mark Lefsrud

**Affiliations:** 1 Bioresource Engineering Department, McGill University, Macdonald Campus, Ste-Anne-De-Bellevue, QC H9X 3V9, Canada; philip.addo@mail.mcgill.ca (P.W.A.);; 2 Bloom Labs, 173 Dr Bernie MacDonald Drive, Bible Hill, NS B6L 2H5, Canada

**Keywords:** antioxidants, *Cannabis sativa*, drying, DPPH, FRAP, *Humulus lupulus*

## Abstract

Efficient determination of antioxidant activity in medicinal plants may provide added value to extracts. The effects of postharvest pre-freezing and drying [microwave-assisted hot air (MAHD) and freeze drying] on hops and cannabis were evaluated to determine the relationship between antioxidant activity and secondary metabolites. The 2,2-diphenyl-1-picrylhydrazine (DPPH) reduction and ferric reducing ability of power (FRAP) assays were assessed for suitability in estimating the antioxidant activity of extracted hops and cannabis inflorescences and correlation with cannabinoid and terpene content. Antioxidant activity in extracts obtained from fresh, undried samples amounted to 3.6 Trolox equivalent antioxidant activity (TEAC) (M) dry matter^−1^ and 2.32 FRAP (M) dry matter^−1^ for hops, in addition to 2.29 TEAC (M) dry matter^−1^ and 0.25 FRAP (M) dry matter^−1^ for cannabis. Pre-freezing significantly increased antioxidant values by 13% (DPPH) and 29.9% (FRAP) for hops, and by 7.7% (DPPH) and 19.4% (FRAP) for cannabis. ANOVA analyses showed a significant (*p* < 0.05) increase in total THC (24.2) and THCA (27.2) concentrations (g 100 g dry matter^−1^) in pre-frozen, undried samples compared to fresh, undried samples. Freeze-drying and MAHD significantly (*p* < 0.05) reduced antioxidant activity in hops by 79% and 80.2% [DPPH], respectively and 70.1% and 70.4% [FRAP], respectively, when compared to antioxidant activity in extracts obtained from pre-frozen, undried hops. DPPH assay showed that both freeze-drying and MAHD significantly (*p* < 0.05) reduced the antioxidant activity of cannabis by 60.5% compared to the pre-frozen samples although, there was no significant (*p* < 0.05) reduction in the antioxidant activity using the FRAP method. Greater THC content was measured in MAHD-samples when compared to fresh, undried (64.7%) and pre-frozen, undried (57%), likely because of decarboxylation. Both drying systems showed a significant loss in total terpene concentration, yet freeze-drying has a higher metabolite retention compared to MAHD. These results may prove useful for future experiments investigating antioxidant activity and added value to cannabis and hops.

## 1. Introduction

Hops (*Humulus lupulus*) possess unique chemical compounds that contribute greatly to the bitterness, flavor, and aroma of beer [1]. Cannabis (*Cannabis sativa*), is a close relative of hops and is predominately cultivated for its medicinal and psychotropic properties [2]. Hops and cannabis both belong to the taxonomy family Cannabaceae and thus have related physiological traits and contain similar secondary metabolites, some of which exhibit antioxidant capacity [3]. Plant antioxidants play important roles in the acclimation or adaptation of plants to a variety of environmental stressors and are beneficial for human health [4]. As part of a balanced nutritional diet, these antioxidants provide protection against damage caused by free radicals involved in the development of many chronic illnesses such as cancer and cardiovascular diseases [5].

Antioxidants are bioactive compounds that, even in small amounts, slow or stop oxidation processes influenced by reactive oxygen species (ROS) or ambient oxygen enzymes [6,7]. Various studies have reported that diverse naturally occurring antioxidants are found in medicinal plants at different concentrations and with varied physical and chemical properties [8,9,10,11,12,13,14,15,16]. Although antioxidants are classified as either lipid-soluble (hydrophobic) and water-soluble (hydrophilic), plant-based antioxidants such as phenolic compounds and vitamin C are mostly hydrophilic [17,18]. Phenolic compounds (terpenes, flavonoids, and carotenoids) in plants act as structural polymers, attractants for insects, ultraviolet protectors, signal compounds, and defense response chemicals [19]. Hydrophobic antioxidants such as carotenoids and vitamin E protect cell membranes from lipid peroxidation [20]. Antioxidants may alternately be classified as enzymatic or non-enzymatic based on their catalytic action [18,21]. Enzymatic antioxidants convert harmful oxidative products via a multi-step enzymatic process, in the presence of cofactors such as copper, zinc, manganese, and iron to stable hydrogen peroxide (H_2_O_2_), converting it to water [22]. Non-enzymatic antioxidants prevent the spread of free radicals [18]. Based on their direct or indirect antioxidant defense mechanism, plant antioxidants can be classified as primary or secondary [15,23]. Primary antioxidants such as catalase act as chain-breaking antioxidants by reacting directly with free radicals [24]. Secondary antioxidants, including glutathione-s-transferase, work indirectly as singlet oxygen quenchers, peroxide decomposers, metal chelators, oxidative enzyme inhibitors and UV radiation absorbers [23]. 

Hops contain α-acids (cohumulene, humulone, and adhumulone), β-acids (colupulene, n-lupulone and adlupulone), and xanthohumol, which are the precursors of bittering agents in beer [25]. Xanthohumol is the major prenylated flavonoid in hops and it is synthesized in glandular trichomes of hop inflorescences [26]. Bitter acids in hops are formed from the acylation of one molecule of acyl-CoA and three molecules of malonyl-CoA to form phlorisovalerophenone [27,28]. Hops may be considered a natural antioxidant since the α-acids, β-acids, and xanthohumol present in this plant have significant hydroxyl radical scavenging and antioxidant activities [26,29,30]. A comparative study using three hop accessions (Calypso, Cascade, and Cluster) demonstrated the presence of high antioxidant activity with the DPPH assay, measuring 342.3, 211.8, and 196.8 µg mL^−1^, respectively [16]. 

Major active secondary compounds found in the cannabis plant are the cannabinoids [31], a group of chemical compounds that alter neurotransmission activity of the brain by acting on the cannabinoid receptors [32,33,34,35,36]. Research studies have shown that cannabinoids exhibit antioxidant properties [37,38,39]. Cannabinoids, like other antioxidants, interrupt free radical chain reactions, chelating free radicals by donating their electrons or hydrogen atom and transforming them into less active forms [18]. Dawidowicz et al. (2021) showed that the degree of antioxidant activity by acidic and neutral cannabinoids can be attributed to the number of phenolic hydroxyl groups in individual cannabinoids. Cannabinolic acid (CBDA) (13.3%) and cannabidiol (CBD) (53.3%) showed significantly (*p* < 0.05) greater scavenging power compared to tetrahydrocannabinolic acid (THCA) and tetrahydrocannabinol (THC), respectively. Hops cannot synthesize cannabinoids as they lack the oxidocyclase enzymes needed to convert cannabigerolic acid (CBGA) to various cannabinoids [40].

Other antioxidant compounds of interest produced by hops and cannabis are terpenes and phenols [9]. Terpenes, or isoprenoids, are one of the largest and most diverse groups in plants [41]. Although terpenes and volatile phenols are mostly responsible for their characteristic aroma, they possess beneficial health benefits such as anticancer, antimicrobial, antifungal, antiviral, analgesic, anti-inflammatory, and antiparasitic activities [42,43,44]. In vitro studies by Rufino et al. (2015) showed that myrcene, one of the most abundant terpenes in hops and cannabis, has significant anti-inflammatory and anti-catabolic properties, and is useful for halting or slowing down cartilage destruction and osteoarthritis progression. Phenolic compounds, including terpenes, are reportedly powerful antioxidants with high scavenging properties [8].

Plant secondary metabolite biosynthesis and antioxidant activity can be disrupted and altered during postharvest storage and drying [45,46,47]. Storage studies by Grafström et al. (2019) over four years showed that CBD is not prone to oxidative degradation and is stable over time, while decarboxylation of THCA to THC which occurs in stored plant material is increased by the presence of oxygen and higher temperatures [48,49]. Specifically, THC concentrations markedly increase from 1.5% to 2.1%, 12.3% and 12.8% when stored at 50 °C, 100 °C, and 150 °C, respectively, due to THCA decarboxylation [45]. Hop buds stored at 20 °C in a dark room showed decreased α-acid concentrations from 186.9 μmol g^−1^ to 37.0 μmol g^−1^ and β-acids from 107.7 μmol g^−1^ to 50.9 μmol g^−1^. Both α-acids and β-acids are oxidized rapidly during hop storage [50]. Decreases in α-acids and β-acids can decrease the antioxidant capacity of hops.

The effects of pre-freezing and drying on hop terpene content has been reported and the optimal conditions for freeze-drying and microwave assisted hot air drying (MAHD) were explored [51]. This previous study showed that the low temperature used during freeze-drying preserved 16.6% to 68.3% of the major terpenes present in hops compared to hot air and MAHD systems, respectively. Pre-freezing caused significant structural damage to hops and this was similarly observed for cannabis in a related trial [51].

The main objective of this follow-up study was to investigate the effects of pre-freezing, prior to drying hops and cannabis, on antioxidant capacity using pre-optimal drying conditions. The suitability and efficiency of 2,2-diphenyl-1-picrylhydrazine (DPPH) and ferric reducing antioxidant power (FRAP) assays were examined and compared for estimating total antioxidant activity (TAC) in hops and cannabis extracts from biomass subjected to these postharvest methods. Given the legislative focus on documenting scientific literature that scrutinizes the therapeutical potential of cannabis for medical use, the relationship between antioxidant capacity and valued secondary metabolites in these two crops was examined.

## 2. Results

### 2.1. Ferric Reducing Antioxidant Power (FRAP) and 2,2-diphenyl-1-picrylhydrazyl (DPPH) Calibration Curves

**Figure 1:**
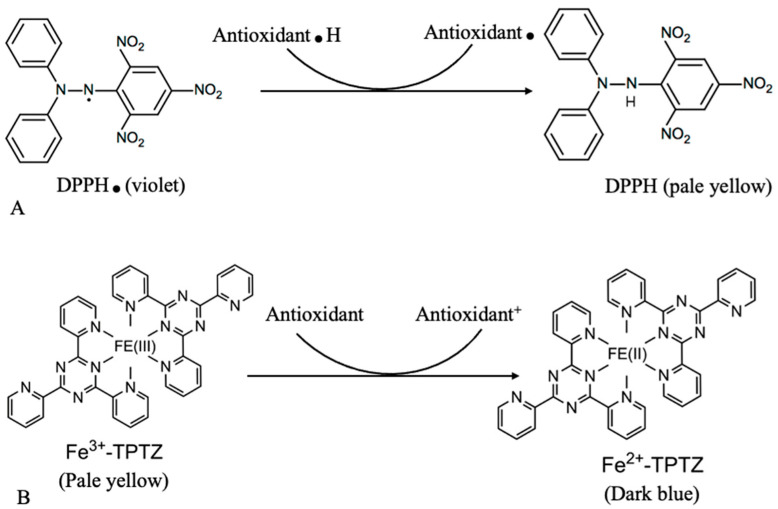
Reaction underlying the (**A**) DPPH (2,2-diphenyl-1-picrylhydrazyl) and (**B**) FRAP (ferric 2,4,6-tri(2-pyridyl)-s-triazine) antioxidant assays.

This study aimed to compare the suitability and efficiency of the DPPH and FRAP assays when measuring total antioxidant activity in hops and cannabis extracts procured from differently processed biomass, including a pre-freezing step followed by freeze-drying or MAHD. The DPPH and FRAP colorimetric assays are universal tools that are currently used for assessing nonenzymatic antioxidants present in plants [15,52]. The DPPH assay measures the radical scavenging activity of most phenolic compounds such as flavonoids and tannins [37,53]. The FRAP assay is a measure of the transition metal ion chelating activity of antioxidants such as ascorbic acid, uric acid and polyphenolic compounds such as catechins under acidic conditions [37,54]. Bleaching of the DPPH solution from violet to pale yellow increases with an increase of antioxidant activity in each sample (Figure 1A). This assay is based on the reduction of the free radical DPPH to DPPH-H. The FRAP assay uses the reduction of ferric ions (Fe^3+^) to ferrous ions (Fe^2+^) as the signal and measures the change in absorbance at 593 nm owing to the formation of a blue colored Fe^2+^-tripyridyltriazine compound from the colorless oxidized Fe^3+^ form by the action of electron-donating antioxidants (Figure 1B). 

**Figure 2:**
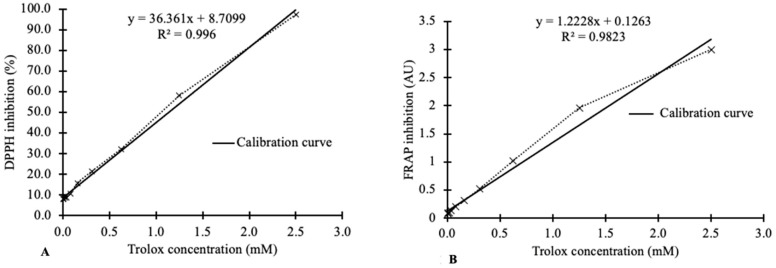
Calibration curves used for (**A**) DPPH (2,2-diphenyl-1-picrylhydrazine) reduction and (**B**) FRAP (reduction of Fe^3+^ to Fe^2+^) assays in the presence of different Trolox concentrations.

The percentage of radical scavenging capacity for different Trolox concentrations used for DPPH and FRAP assay calibration curves is shown in Figure 2. Figure 2 shows inhibition values using 0.005 to 2.5 mM Trolox concentrations. Preliminary data exhibited a flattening of the graph between 2.5 and 10 mM Trolox concentration. This can be attributed to the almost complete quenching of DPPH and FRAP by Trolox, which does not affect the absorbance values. As such, sample dilution is necessary to dilute samples to within the measurable range (0.005 to 2.5 mM Trolox concentrations). A similar curve flattening observation was made by Sochor et al. (2010) and Pisoschi et al. (2009), where the absorbance of Trolox did not change at concentrations of 200–1000 μmol L^−1^ and 0.15–0.2 mM, respectively. Calibration graphs (Figure 2) used to quantify the antioxidant capacities of hops and cannabis in this study are linear, in the range 0.005 to 2.5 mM for Trolox, with strong correlation coefficients (R^2^) of 0.996 and 0.982 for DPPH and FRAP, respectively.

### 2.2. Antioxidant Activity of Hops and Cannabis

**Figure 3:**
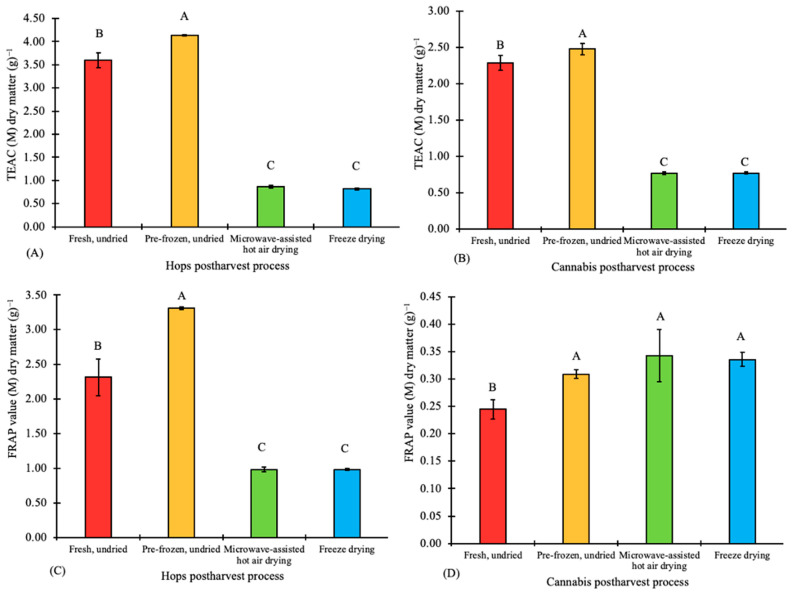
Total antioxidant activity in extracts from hops (*Humulus lupulus*) and cannabis (*Cannabis sativa*) inflorescences using the DPPH (**A**,**B**) and FRAP (**C**,**D**) assays. Bars with the same letter are not significantly (*p* < 0.05) different. DPPH: 2,2-diphenyl-1-picrylhydrazine; FRAP: Ferric reducing antioxidant power; TEAC: Trolox equivalent antioxidant activity.

The observed TEAC and FRAP values determined in hops and cannabis are presented in Figure 3. The antioxidant activity of extracts derived from fresh, untreated hops was 3.6 TEAC (M) dry matter^−1^ and 2.32 FRAP (M) dry matter^−1^. Extracts from fresh, untreated cannabis samples had 2.29 TEAC (M) dry matter^−1^ and 0.25 FRAP (M) dry matter^−1^ antioxidant values. The lower antioxidant activity observed in cannabis relative to hops can be attributed to the presence of α-acids and β-acids in hops [25]. Analysis of variance tests showed that pre-freezing, freeze-drying and MAHD significantly affected (*p* < 0.05) the antioxidant activity of hops and cannabis when evaluated with the DPPH and FRAP assays.

Pre-freezing the hops and cannabis samples before drying increased the antioxidant values by 13% (DPPH assay) and 29.9% (FRAP assay) for hops, and by 7.7% (DPPH assay) and 19.4% (FRAP assay) for cannabis (Figure 3). DPPH assays used for this study show that freeze-drying and MAHD significantly (*p* < 0.05) reduced the antioxidant activity in hops by 79% and 80.2%, respectively, compared to pre-frozen, undried samples. A similar observation was made for hops using the FRAP assay, as antioxidant activity was reduced by 70.1% and 70.4% under freeze-drying and microwave-assisted hot air drying, respectively, when compared to pre-frozen, undried hops. Both freeze-drying and MAHD significantly (*p* < 0.05) reduced the antioxidant activity of cannabis by 60.5% using the DPPH assay. However, there was no significant (*p* < 0.05) difference between the antioxidant activity values for pre-frozen, freeze-dried and microwave-assisted hot air dried cannabis samples using the FRAP method. 

### 2.3. Cannabinoid and Terpenes in Hops and Cannabis

**Figure 4:**
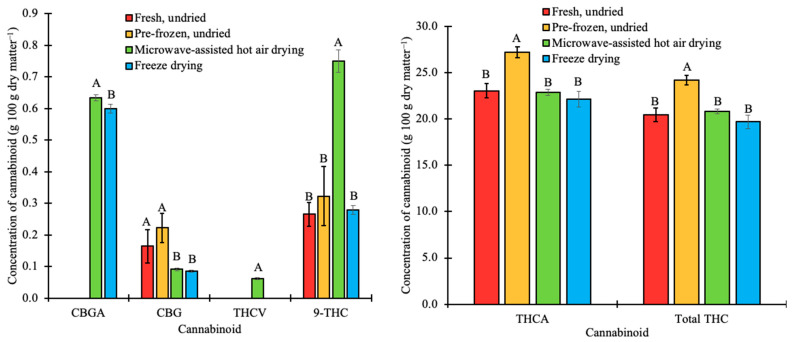
Concentration of cannabinoids in pre-frozen undried, microwave-assisted hot air dried, and freeze dried-cannabis (*C. sativa*) biomass. Bars with the same letter are not significantly (*p* < 0.05) different.

For a comparison of different postharvest treatments and valued phytochemicals in extracted hops and cannabis extra inflorescences, total THC content and major cannabinoid concentrations (tetrahydrocannabinolic acid [THCA], tetrahydrocannabinol [Δ9-THC], tetrahydrocannabivarin [THCV], cannabigerolic acid [CBGA], and cannabigerol [CBG]) in *C. sativa* were determined (Figure 4). In the same figure, the cannabinoid and terpene content in extracts obtained from fresh, undried cannabis samples were compared to cannabinoids and terpene content in extracts obtained in this study. CBDA, CBD, and total CBD content are not presented, as the concentration of CBDA and CBD was below the limit of detection of the instrumentation and methodology. Extracts from fresh, undried *Cannabis sativa* had total THC, THCA, THC, and CBG concentrations of 20.5 g 100 g dry matter^−1^, 23.1 g 100 g dry matter^−1^, 0.27 g 100 g dry matter^−1^, and 0.16 g 100 g dry matter^−1^, respectively. ANOVA analyses showed a significant (*p* < 0.05) increase in the total THC (24.2 g 100 g dry matter^−1^) and THCA (27.2 g 100 g dry matter^−1^) concentrations in extracts obtained from pre-frozen, undried samples compared to fresh, undried samples. However, there was no significant (*p* < 0.05) increase in THC (0.32 g 100 g dry matter^−1^) and CBG (0.22 g 100 g dry matter^−1^) concentrations in extracts from the pre-frozen, undried samples compared to the fresh, undried samples. 

The concentration of CBGA measured herein was below the limit of detection of the instrumentation and methodology in the extracts of fresh and pre-frozen, undried samples, likely because CBGA serves as the precursory molecule to the other cannabinoids [55]. Various bioengineering studies have demonstrated that the prenylation of olivetolic acid (OA) by geranyl diphosphate (GPP) to form a CBGA is an anabolic process [35,56,57]. Hence, the observed increase in the average concentration of CBGA to 0.63 g 100 g dry matter^−1^ (MAHD-dried samples) and 0.6 g 100 g dry matter^−1^ (freeze-dried samples) can be attributed to the high drying temperatures used. Recent published reviews of the cannabis post-harvest processing methods [49,58] indicate that with the application of heat, THCA and THCVA change into their active forms of THC and THCV, respectively. Compared to the fresh and pre-frozen, undried samples, extracts from MAHD biomass had significantly (*p* < 0.05) greater THC content by 64.7% and 57%, respectively. ANOVA analyses show that the change in THCA and THC in freeze-dried samples compared to the fresh and pre-frozen, undried samples was not significant (*p* < 0.05). 

**Figure 5:**
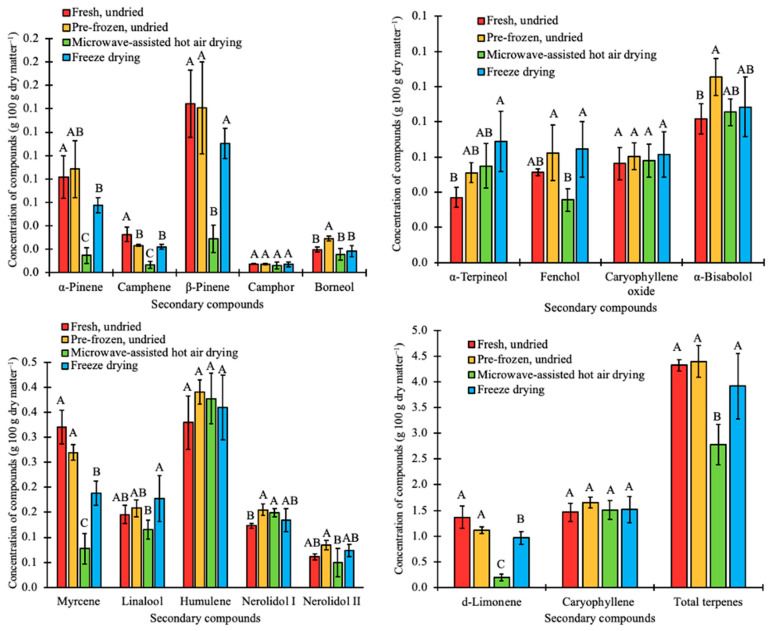
Concentration of terpenes in cannabis in fresh undried, pre-frozen undried, microwave-assisted hot air dried, and freeze-dried cannabis (*C. sativa*) biomass. Bars with the same letter are not significantly (*p* < 0.05) different.

**Figure 6:**
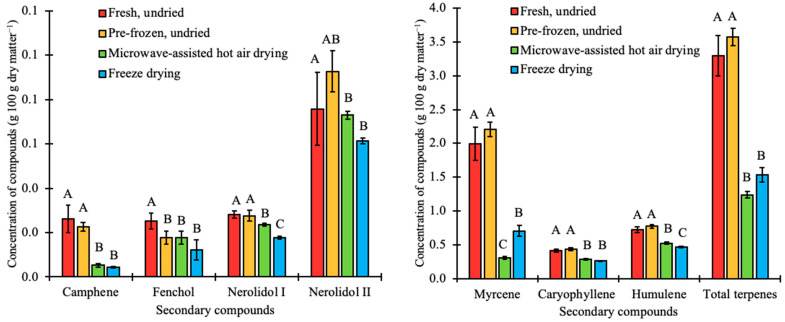
Concentration of terpenes in hops in fresh undried, pre-frozen undried, microwave-assisted hot air dried, and freeze-dried hops (*H. lupulus*) biomass. Bars with the same letter are not significantly (*p* < 0.05) different.

A total of 16 and 7 terpene compounds were identified in the cannabis and hop samples, respectively. All seven terpene compounds identified in hops were present in cannabis at different concentrations. Despite the major differences in secondary compounds in cannabis and hops used for the study, the main terpenes were myrcene, caryophyllene, and humulene. These provide the inflorescence with a peppery, citrus, and hoppy mixed aroma [41,59]. The caryophyllene concentration in cannabis was 71.2% greater than that of hops. However, humulene had a higher concentration (54.8%) in hops compared to cannabis. Data represented in Figure 5 and Figure 6 indicate that the concentration of myrcene in fresh, undried hops was reduced from 1.9 to 0.3 g 100 g dry matter^−1^ (MAHD) and to 0.7 g 100 g dry matter^−1^ (freeze-dried) and for fresh, undried cannabis, the concentration reduced from 0.3 to 0.1 g 100 g dry matter^−1^ (MAHD) and to 0.2 g 100 g dry matter^−1^ (freeze-dried). Rajkumar et al. (2017) showed that compared to fresh, undried carrots, myrcene was reduced from 2.3 to 0.4 g 100 g dry matter^−1^ (MAHD) and to 1.6 g 100 g dry matter^−1^ (FD). This shows that freeze-drying resulted in a higher terpene retention compared to MAHD for these crops.

Major terpene content was similarly determined and compared for cannabis and hops subjected to the same postharvest drying conditions (Figure 5 and Figure 6). The average total terpene content from fresh, undried cannabis and hop samples was 4.3 g 100 g dry matter^−1^ and 3.3 g 100 g dry matter^−1^, respectively. ANOVA analyses showed that the increase in the total terpene content to 4.4 g 100 g dry matter^−1^ and 3.6 g 100 g dry matter^−1^ for cannabis and hops, respectively, by pre-freezing was not significant (*p* < 0.05). For freeze-dried and microwave-assisted hot air dried hop samples (Figure 6), the average total terpene significantly (*p* < 0.05) reduced to 1.5 g 100 g dry matter^−1^ and 1.2 g 100 g dry matter^−1^, respectively. However, freeze drying preserved the total terpenes (3.9 g 100 g dry matter^−1^) in cannabis samples compared to microwave-assisted hot air drying (2.8 g 100 g dry matter^−1^) (Figure 5). The high temperature used during MHAD significantly (*p* < 0.05) reduced total terpene content in the fresh, undried samples from 4.3 to 2.8 g 100 g dry matter^−1^. Terpenes evaporate easily in MAHD since the cannabis and hop structures and dimensions permit its evaporation even at 35 °C, while freeze-drying uses a relatively very low temperature which limits the evaporation of terpenes [60]. Hence, freeze-drying, rather than hot-air drying, is recommended to help preserve terpenes in hops and cannabis during postharvest processing.

## 3. Discussion

Antioxidants may be hydrophobic (lipid-soluble) and hydrophilic (water-soluble) substances, yet plant-based antioxidants are mostly hydrophilic [18,61]. Results obtained in this study showed an increased antioxidant activity in the pre-frozen samples, which can be attributed to the structural damage caused by the ice crystal formation reported previously in a preceding study; scanning electron microscopy analyses of cannabis samples showed that the cold temperature used during pre-freezing and consequent ice crystal formation caused wrinkling of cannabis trichome stalks and cannabis trichome heads to fall off [51]. Other research has shown that pre-freezing exerts positive effects on the quality and functional properties of plant material since a frozen state allows the release of bioactive compounds as bound phenolic acids and anthocyanins, resulting in increased antioxidant activity [13,62]. Leong and Oey (2012) showed that pre-freezing apricots (*Prunus armeniaca*) at −20 °C increased the concentration of vitamin C and β-carotene by 55.5% and 10.7%, respectively.

The high temperature used during MAHD and freeze-drying caused a significant (*p* < 0.05) reduction in the antioxidant activity in both hops and cannabis using the DPPH assay compared to the pre-frozen samples. This can be attributed to a reduction in free phenolic compounds present in the samples, as DPPH measures the scavenging activity of phenolic compounds [11,30]. Lang et al. (2019) observed a significant (*p* > 0.05) reduction (5.7%) in the total free phenolic compounds in rice (*Oryza sativa*) when the drying temperature was increased from 20 °C to 80 °C. Significant (*p* < 0.05) differences were not observed between the antioxidant activity values for pre-frozen, freeze-dried and MAHD-dried cannabis samples using the FRAP method, likely due to the presence of iron-chelating compounds such as cannabinoids in the cannabis extract samples (Figure 3). Cannabinoids can interfere with the FRAP assay by chelating the Fe^3+^ irons in the FRAP reagent mixture; Dawidowicz et al. (2021) showed that cannabinoids are antioxidant agents as they can scavenge free radicals, and THC’s antioxidant activity was greater by 35.3% with the FRAP assay when compared to DPPH assay. Given these data, the FRAP assay is recommended for determining antioxidant activity in cannabis and hop inflorescences. Further studies using other antioxidant activity assays such as oxygen radical absorbance capacity (ORAC) and determining the presence of antioxidants in different cannabis and hop plant organs could be explored.

The significant (*p* < 0.05) increase in total THC and THCA concentrations can be attributed to the pre-freezing step. Pre-freezing causes structural damage to trichome structures and can be considered as an abiotic stressor [51,63]. Ahmed et al., (2013) reported that abiotic stresses increased total phenolic compounds (TPC) by 62.5% in barley (*Hordeum vulgare*) compared to the control upon harvest. Taking this into account, it is plausible that the structural damage incurred by trichomes during pre-freezing step helps release trapped secondary metabolites.

Cannabinoid analyses showed a significant (*p* < 0.05) increase in THC for MAHD-dried samples compared to fresh, undried (64.7%) and pre-frozen, undried samples (57%). This can be explained by the non-enzymatic decarboxylation process [49]. However, freeze-drying did not cause a significant change in the concentration of THC and THCA in the samples. Hence, freeze-drying can be used to preserve the secondary metabolites present in cannabis and these data support previous findings [51]. These findings are comparable to other crops preserved in this manner [64,65]. Moreno et al. (2020) showed that the non-enzymatic decarboxylation of acidic cannabinoids to neutral cannabinoids increases with the increase in temperature. Using a decarboxylation time of 60 min, the concentration of THC increased from 0.02 g 100 g dry matter^−1^ (80 °C) to 0.03 mg g dry matter^−1^ (120 °C). Similar observations were made for the terpenes present in hops and cannabis. MAHD caused significant (*p* > 0.05) thermal degradation of terpenes in the studied samples.

## 4. Materials and Methods

### 4.1. Sample Preparation

Hops (Brewer’s gold) were cultivated outdoors at McGill University’s Macdonald Campus farm in Sainte-Anne-de-Bellevue, QC, Canada. Hops were planted on 3 May 2022, and harvested from mid-September to the end of October 2022. Preliminary tests were conducted using a split plot design to limit the differences between the hops harvested from the different plots. The cannabis inflorescence was harvested from an indoor-grown accession (Qrazy Train). Harvested hops and cannabis biomass was pre-frozen at −20 °C for a minimum of 24 h prior to drying and analysis as described previously [51]. The initial moisture content of the hops and cannabis inflorescence was determined using a hot air oven (Fisher Scientific 6903 Isotemp oven, Waltham, MA, USA). Each sample was dried at 50 °C for 24 h.

### 4.2. Freeze Drying of Hops and Cannabis

Optimal freeze-drying conditions for cannabis and hop biomass identified previously were applied to this experiment [51]. For each condition, approximately 100 g pre-frozen cannabis and hop inflorescence samples were placed in plastic trays and transferred to a laboratory-scale vacuum freeze-dryer (Martin Christ Gefriertrocknungsanlagen GmbH Gamma 1–16 LSCplus, Osterode, Lower Saxony, Germany) with a condenser temperature of −55 °C. Freeze-drying was carried out at 20 °C for 24 h at 0.85 mbar until the sample reached a dry basis moisture content of 12%. Dried samples were transferred into a food-grade plastic bag and stored in a refrigerator at 5 °C before analyses. Each experiment was performed in triplicate using three different biomass samples.

### 4.3. Microwave-Assisted Hot Air Drying of Hops and Cannabis (MAHD)

Optimal MAHD conditions for cannabis and hop biomass identified previously were applied to this experiment [51]. MAHD was conducted in an automated laboratory-scale microwave oven with several modifications. Briefly, the main components were a 2450 MHz microwave generator (Gold Star 2M214, Seoul, South Korea) with adjustable power (0 to 750 W), waveguides, a three-port circulator, a manual three-stub tuner to match the load impedance, microwave couplers to measure forward and reflected power, a carbon load to absorb reflected power, and a microwave cavity made of brass (0.47 × 0.47 × 0.27 m) in which the samples were processed. In each experiment, approximately 100 g pre-frozen hops and cannabis inflorescence were placed in a nylon mesh sample holder tray (diameter = 0.21 m). The plant material was spread in one layer and placed inside the microwave cavity. Drying was performed until the sample reached a dry basis moisture content of 12%. Dried samples were transferred into a plastic bag and stored in a refrigerator at 5 °C before analyses. Drying was performed in triplicate under each condition.

### 4.4. Extraction of Secondary Metabolites

Representative samples for each of the drying conditions and fresh samples were immersed in liquid nitrogen before grinding with a coffee grinder (Hamilton Beach, Belleville, ON, Canada). Ground samples were allowed to equilibrate to room temperature before 0.75 g was weighed in a 50 mL Falcon tube and recorded. Each sample was allowed to sit for 10 min on the scale (Mettler AE50 analytical balance, Columbus, Ohio, United States of America) until there was <1 mg change in mass. This is done to ensure that most of the liquid nitrogen had evaporated from the sample and the proper sample mass was obtained. For the extraction of secondary metabolites, 20 mL high-pressure liquid chromatography (HPLC)-grade methanol (Thermo Fisher Scientific, Waltham, MA, USA) was added to each Falcon tube and vortexed (Thermo Scientific vortex, Waltham, MA, USA) for 20 min at 500 rpm. Each sample was filtered using Whatman™ filter paper (Thermo Fisher Scientific, Waltham, MA, USA) and allowed to filter for 20 min. Residual cannabis biomass was placed into a new 50 mL Falcon tubes and subjected to a second extraction process to ensure 99.5% of the secondary metabolites were extracted. The second extract was added to the corresponding first extract, resulting in a 40× dilution total extract. 

### 4.5. Measuring Antioxidant Activity with the 2,2-diphenyl-1-picrylhydrazyl (DPPH) Assay

Antioxidant activities of hops and cannabis were determined using the DPPH assay introduced by Brand-Williams et al. (1995) and used by Dawidowicz et al. (2021) for cannabis, with some modifications. A calibration curve was generated using different serial dilutions of a 10 mM Trolox^®^ standard (Sigma-Aldrich, Saint Louis, MI, USA) in HPLC-grade methanol (Thermo Fisher Scientific, Waltham, MA, USA). A stock solution of 0.1 mM DPPH ion (Sigma-Aldrich, Saint Louis, MA, USA) in HPLC-grade methanol was prepared fresh daily. Aliquots (100 μL) of extracted samples or standards were placed in 15 mL Falcon tubes and 2900 μL of DPPH ion stock solution was added. The mixture was subjected to vigorous vortexing (Thermo Scientific vortex, Waltham, MA, USA) for 30 sec then incubated for 30 min at room temperature in the dark. Absorbances were measured at 517 nm using the Ultropec 2100 pro ultraviolet/visible spectrophotometer (Biochrom Limited, Cambridge, England). A DPPH ion solution was used as a control and HPLC-grade methanol was used to zero the spectrophotometer. The average radical scavenging activity of the samples was calculated and the DPPH inhibition (%) was calculated using Equation (1). Concentration (M) of Trolox equivalent antioxidant activity (TEAC) using the calibration curve was calculated using Equation (2). Results are reported as the concentration (M) of Trolox equivalent antioxidant activity (TEAC) per gram dry matter sample using Equation (3). The experiment was carried out in triplicate.

(1) $$\%\;\mathrm{DPPH}\;\mathrm{inhibition}=\frac{{\mathrm{Absorbance}}_{\mathrm{control}}-{\mathrm{Absorbance}}_{\mathrm{sample}}}{{\mathrm{Absorbance}}_{\mathrm{control}}}$$

(2) $$\mathrm{TEAC}\;\left(M\right)=\frac{\left(\frac{\%\;\mathrm{DPPH}\;\mathrm{inhibition}-8.7009}{36.361}\right)}{1000}$$

(3) $$\mathrm{TEAC}\;\left(M\right)\;\mathrm{dry}\;\mathrm{matter}\;{\left(g\right)}^{-1}=\frac{\mathrm{Extraction}\;\mathrm{volume}\;\left(0.04\;L\right)\times \;\mathrm{TEAC}\;\left(M\right)}{\mathrm{Analysis}\;\mathrm{volume}\;\left(0.0001\;L\right)\times \left(\mathrm{sample}\;\mathrm{mass}\;-\left(\%\;\mathrm{mc}\times \mathrm{sample}\;\mathrm{mass}\right)\right)}$$

### 4.6. Measuring Antioxidant Activity with the Ferric Reducing Antioxidant Power (FRAP) Assay

The antioxidant capacity of hops and cannabis was additionally determined using the ferric reducing antioxidant power (FRAP) assay based on methods developed by Benzie and Strain (1996) and Dawidowicz et al. (2021) for cannabis, with some modifications. The standard curve was prepared using different serial dilution concentrations (10–0.004 mM) of Trolox (Sigma-Aldrich, Saint Louis, MI, USA). The FRAP reagent was prepared from 300 mM sodium acetate buffer (pH 3.6), 20 mM 2,4,6-tri(2-pyridyl)-s-triazine (TPTZ) (Sigma-Aldrich, Saint Louis, MI, USA) solution in 40 M hydrochloric acid (Thermo Fisher Scientific, Waltham, MA, USA) and 20 mM ferric chloride (FeCl_3_) (Sigma-Aldrich, Saint Louis, MI, USA) solution in proportions of 10:1:1 (*v*/*v*), respectively. The FRAP solution was prepared fresh daily and warmed to 37 °C in a water bath for 10 min prior to use. An aliquot (100 μL) of extracted samples or standards was placed in 15 mL Falcon tubes and 2900 μL FRAP stock solution was added. After vigorous vortex (Thermo Scientific vortex, Waltham, MA, USA) for 30 sec, the mixture was incubated for 60 min at room temperature and in darkness. Absorbances were measured at 593 nm using the Ultropec 2100 pro ultraviolet/visible spectrophotometer (Biochrom Limited, Cambridge, England). The FRAP solution was used as a control and HPLC-grade methanol was used to zero the spectrophotometer. The experiment was carried out in triplicate. FRAP inhibition was calculated using Equation (4). The FRAP value (antioxidant activity) was calculated using the calibration curve and Equation (5). Results are reported as FRAP value (M) per gram dry matter sample using Equation (6).

(4) $$\mathrm{FRAP}\;\mathrm{inhibition}\;\left(\mathrm{AU}\right)={\;\mathrm{Absorbance}}_{\mathrm{sample}}-{\;\mathrm{Absorbance}}_{\mathrm{control}}$$

(5) $$\mathrm{FRAP}\;\mathrm{value}\;\left(M\right)=\frac{\left(\frac{\mathrm{FRAP}\;\mathrm{inhibition}-0.1263}{1.2228}\right)}{1000}$$

(6) $$\mathrm{FRAP}\;\mathrm{value}\;\left(M\right)\;\mathrm{dry}\;\mathrm{matter}\;{\left(g\right)}^{-1}=\frac{\mathrm{Extraction}\;\mathrm{volume}\;\left(0.04\;L\right)\times \;\mathrm{FRAP}\;\mathrm{value}\;\left(M\right)}{\mathrm{Analysis}\;\mathrm{volume}\;\left(0.0001\;L\right)\times \left(\mathrm{sample}\;\mathrm{mass}\;-\left(\%\;\mathrm{mc}\times \mathrm{sample}\;\mathrm{mass}\right)\right)}$$

### 4.7. Cannabinoid Analyses

Waters Acquity Ultra High-Performance Liquid Chromatography (UPLC) with a tunable ultraviolet (TUV) detector (Waters™, Mississauga, ON, Canada) was used for cannabinoid analyses. Each extract was further diluted 50× (for analysis of major cannabinoids) and 4× (for analysis of minor cannabinoids and terpenes) using HPLC-grade methanol (Thermo Fisher Scientific, Waltham, MA, USA). One-milliliter samples of each extract were pipetted into HPLC vials for cannabinoid analysis. The Waters cortex column was used to separate cannabinoids with a sample injection volume of 2 μL and a column temperature of 30 °C, equipped with an isocratic gradient pump. Mobile phase A consisted of 22% reverse osmosis water and 0.1% formic acid (Sigma-Aldrich, Saint Louis, MI, USA). HPLC-grade acetonitrile (78%) (Thermo Fisher Scientific, Waltham, MA, USA) was used for mobile phase B. Quantification of the cannabinoids was performed using an external calibration curve developed using 7 standard cannabinoids (LGC standards, Manchester, NH, USA and Sigma Aldrich, Saint Louis, MI, USA).

### 4.8. Terpene Analysis

Terpene analysis assay previously described by Addo et al. (2022) was used for this study. Gas chromatography-tandem mass spectrometer was used for terpene analyses. One-milliliter samples of each extract were pipetted into gas chromatograph (GC) vials for terpene analysis. Separation of the terpenes was performed with an Agilent 7820A GC coupled to an Agilent 7693 autosampler and a flame ionization detector (FID) (Agilent Technologies, Mississauga, Ontario, Canada). The system was equipped with an injector containing a capillary column (30 m × 250 μm × 0.25 μm nominal Agilent Technologies DB-5 Model) using split injection (ratio 50:1) with a hydrogen carrier gas (40 mL min^−1^). An injection volume of 5 μL each sample with a 10 μL syringe size was used. The oven temperature of the mass spectrometer was initially programmed at 35 °C and held for 4 min. The temperature was increased at a rate of 10 °C min^−1^ up to 105 °C held for 0 min, increased at a rate of 15 °C min^−1^ up to 205 °C held for 0 min, and lastly increased at a rate of 35 °C min^−1^ up to 270 °C held for 5 min. The inlet temperature into the FID detector was set at 340 °C. Spectra were recorded at three scans from 50 m z^−1^ to 400 m z^−1^. The ionization mode was used with an electronic impact at 70 eV. Quantification of the terpenes was performed using an external calibration of 37 terpenes mostly found in cannabis (LGC standards, Manchester, NH, USA and Sigma-Aldrich, Saint Louis, MI, USA).

### 4.9. Statistical Analysis

All experimental determinations were performed in triplicate. Results from chemical analysis were expressed as average ± standard deviation and were calculated by MS Excel. Statistical analyses were conducted using the JMP software (JMP 4.3 SAS Institute Inc., Cary, NC, USA) with a confidence level (*p* < 0.05) of 95%. Pairwise comparisons of means were carried out using the Student’s statistical t-test. The analysis of the independent variables’ effect (pre-freezing, drying systems, and antioxidant assays) on the dependent variables (antioxidants activity, cannabinoids, and terpenes) was assessed using JMP software. The least-square multiple regression method was used to evaluate the relationship between the independent and dependent variables. Analyses of variance (ANOVA) was carried out to evaluate whether there were significant differences (*p* < 0.05) amongst the samples.

## 5. Conclusions

The effects of postharvest processing on hops and cannabis were evaluated to determine the relationship between antioxidant capacity and secondary metabolites. The study compared the efficiency of DPPH and FRAP assays to estimate total antioxidant activity in hops and cannabis extracts. The antioxidant activity of extracts derived from fresh, untreated samples were 3.6 TEAC (M) dry matter^−1^ and 2.32 FRAP (M) dry matter^−1^ for hops, and 2.29 TEAC (M) dry matter^−1^ and 0.25 FRAP (M) dry matter^−1^ for cannabis. The results showed that although freezing of inflorescences is a preservation technique, pre-freezing the hops and cannabis samples before drying increased the antioxidant values by 13% (DPPH assay) and 29.9% (FRAP assay) for hops, and by 7.7% (DPPH assay) and 19.4% (FRAP assay) for cannabis. Data showed that freeze-drying and MAHD significantly (*p* < 0.05) reduced the antioxidant activity in hops by 79% and 80.2% [DPPH], respectively, and 70.1% and 70.4% [FRAP], respectively, compared to pre-frozen, undried hops. For cannabis, the DPPH assay showed that both freeze-drying and MAHD significantly (*p* < 0.05) reduced the antioxidant activity of cannabis. However, there was no significant (*p* < 0.05) difference between the antioxidant activity values for pre-frozen, freeze-dried, and MAHD cannabis samples using the FRAP method because of the presence of iron-chelating cannabinoids in the cannabis. Results showed that the FRAP assay accurately determines the antioxidant activities of cannabinoids compared to the DPPH assay and is a valuable assay for the cannabis industry. ANOVA analyses showed a significant (*p* < 0.05) increase in the total THC (24.2 g 100 g dry matter^−1^) and THCA (27.2 g 100 g dry matter^−1^) concentrations in pre-frozen, undried samples compared to fresh, undried samples. Non-enzymatic decarboxylation was observed by the significant (*p* < 0.05) increase in the THC in MAHD-dried samples compared to fresh, undried (64.7%) and pre-frozen, undried (57%). Although both drying systems showed a significant loss in the total terpene concentration, freeze-drying has higher terpene retention compared to MAHD. Freeze drying should be used as the drying system for medicinal plants to reduce the postharvest losses of secondary metabolites and decarboxylation of cannabinoids.

## Data Availability

Not applicable.
